# Optimal postoperative pain management after VATS lung resection by thoracic epidural analgesia, continuous paravertebral block or single-shot intercostal nerve block (OPtriAL): study protocol of a three-arm multicentre randomised controlled trial

**DOI:** 10.1186/s12893-022-01765-y

**Published:** 2022-09-04

**Authors:** L. N. Spaans, M. G. W. Dijkgraaf, P. Meijer, J. Mourisse, R. A. Bouwman, A. F. T. M. Verhagen, F. J. C. van den Broek, OPtriAL study group, Denis Susa, Eino van Duyn, Jan-Willem Potters, Erik de Loos, Herman Rijna, Annemieke Boom, Marieke Kuut, Nike Hanneman, Jelle Bousema, Renee van den Broek, Valentin Noyez, Jeroen Hendriks, Chris Dickhoff

**Affiliations:** 1 Department of Surgery, Máxima Medical Center, Veldhoven, The Netherlands; 2 University of Amsterdam, Amsterdam, The Netherlands; 3 Department of Epidemiology and Data Science, Amsterdam University Medical Centers, University of Amsterdam, Amsterdam, The Netherlands; 4 Department of Anesthesiology, Máxima Medical Center, Veldhoven, The Netherlands; 5 Department of Anesthesiology, Radboud University Medical Center, Nijmegen, The Netherlands; 6 Department of Anaesthesiology, Catharina Hospital, Eindhoven, The Netherlands; 7 Department of Cardiothoracic Surgery, Radboud University Medical Center, Nijmegen, The Netherlands

**Keywords:** Postoperative pain, VATS, Locoregional anaesthesia, Paravertebral block, Thoracic epidural, Intercostal nerve block, ERATS

## Abstract

**Background:**

Adequate pain control after video-assisted thoracoscopic surgery (VATS) for lung resection is important to improve postoperative mobilisation, recovery, and to prevent pulmonary complications. So far, no consensus exists on optimal postoperative pain management after VATS anatomic lung resection. Thoracic epidural analgesia (TEA) is the reference standard for postoperative pain management following VATS. Although the analgesic effect of TEA is clear, it is associated with patient immobilisation, bladder dysfunction and hypotension which may result in delayed recovery and longer hospitalisation. These disadvantages of TEA initiated the development of unilateral regional techniques for pain management. The most frequently used techniques are continuous paravertebral block (PVB) and single-shot intercostal nerve block (ICNB). We hypothesize that using either PVB or ICNB is non-inferior to TEA regarding postoperative pain and superior regarding quality of recovery (QoR). Signifying faster postoperative mobilisation, reduced morbidity and shorter hospitalisation, these techniques may therefore reduce health care costs and improve patient satisfaction.

**Methods:**

This multi-centre randomised study is a three-arm clinical trial comparing PVB, ICNB and TEA in a 1:1:1 ratio for pain (non-inferiority) and QoR (superiority) in 450 adult patients undergoing VATS anatomic lung resection. Patients will not be eligible for inclusion in case of contraindications for TEA, PVB or ICNB, chronic opioid use or if the lung surgeon estimates a high probability that the operation will be performed by thoracotomy. Primary outcomes: (1) the proportion of pain scores ≥ 4 as assessed by the numerical rating scale (NRS) measured during postoperative days (POD) 0–2; and (2) the QoR measured with the QoR-15 questionnaire on POD 1 and 2. Secondary outcome measures are cumulative use of opioids and analgesics, postoperative complications, hospitalisation, patient satisfaction and degree of mobility.

**Discussion:**

The results of this trial will impact international guidelines with respect to perioperative care optimization after anatomic lung resection performed through VATS, and will determine the most cost-effective pain strategy and may reduce variability in postoperative pain management.

*Trial registration* The trial is registered at the Netherlands Trial Register (NTR) on February 1st, 2021 (NL9243). The NTR is no longer available since June 24th, 2022 and therefore a revised protocol has been registered at ClinicalTrials.gov on August 5th, 2022 (NCT05491239). *Protocol version*: version 3 (date 06-05-2022), ethical approval through an amendment (see ethical proof in the Study protocol proof).

**Supplementary Information:**

The online version contains supplementary material available at 10.1186/s12893-022-01765-y.

## Background

Thoracic surgery is associated with significant postoperative pain and discomfort. Adequate pain control after video-assisted thoracoscopic surgery (VATS) for lung resection is important to improve postoperative mobilisation and recovery, and to prevent postoperative pulmonary complications. Moreover, adequate acute postoperative pain control reduces the incidence of (neuropathic) chronic pain [1]. For an enhanced recovery after thoracic surgery (ERATS) the European Society of Thoracic Surgery (ESTS) recommends a combination of multimodal enteral and parenteral analgesia with regional analgesia or local anaesthetic techniques while attempting to avoid opioids and their side effects [2].

Thoracic epidural analgesia (TEA) is the usual care for postoperative pain management following VATS. The central neuraxial block by TEA produces highly effective pain relief and anaesthesiologists are experienced with this technique. Up to date, TEA is the preferred technique by the vast majority of Dutch thoracic surgeons [3]. However, the adverse effects of TEA are clear. Failure rates are 9–30% [4–6] and awake placement is stressful for patients. In addition, TEA is associated with patient immobilisation, bladder dysfunction and hypotension [7].

The disadvantages of TEA initiated the development of unilateral regional techniques for pain management. Single-shot and continuous paravertebral, intercostal, serratus anterior and erector spinae blocks have shown to be safe and effective [8]. A meta-analysis on single-shot versus continuous peripheral nerve blockade showed improved pain control, decreased need for opioids and greater patient satisfaction with the continuous infusion technique [9]. Another non-systematic review suggests poorer pain control in single shot techniques, however, these techniques are easy to perform and have low costs compared to the standard TEA care [10] and lower incidence of adverse events [11]. Regarding postoperative nausea and vomiting and length of stay, locoregional techniques performed better. Unilateral regional techniques are not associated with patient immobilisation, bladder dysfunction and hypotension [12]. Lastly, recent PROSPECT guidelines do not recommend the use of thoracic epidural after VATS based on a Delphi consensus, however, the patient population was not specifically VATS anatomical lung resection [13] and this recommendation is not widely accepted due to lack of convincing evidence [14].

Since no consensus exists on optimal postoperative pain management after VATS lung resection, different ERATS protocols lack unambiguity [15]. Five protocols all used different techniques for postoperative pain management: oral, intravenous, intercostal, paravertebral and epidural anaesthesia.

In addition to pain relief, also other aspects of postoperative recovery are crucial in deciding which analgesic technique is superior. Research on pain assessment continues to be a challenge due to its subjective nature and relation to various outcomes related to recovery. Therefore, anaesthesia and pain experts strongly recommend using a validated patient related outcome measure reporting overall quality of recovery (QoR) [16, 17]. Thus far, only few authors reported [18, 19] about pain and QoR after VATS using the QoR-40 item questionnaire. Recently, Stark and colleagues [20] developed a QoR-15 item questionnaire, which is proven to be an easy to use short version and is a validated and relevant tool for measuring QoR. The QoR-15 questionnaire contains the most relevant questions regarding physical and mental well-being after surgery and focuses on the following five domains: pain, physical comfort, physical independence, psychological support and emotional state.

We designed a multi-centre randomised controlled trial to determine which analgesic technique performs best in an ERATS setting regarding pain and quality of recovery. Since continuous paravertebral block and single-shot intercostal nerve block are the most popular loco-regional analgesic techniques after VATS lung resection [3, 21], we selected these two techniques as the intervention techniques to be compared with TEA.

## Methods/design

### Hypothesis

Postoperative pain management by using either continuous paravertebral block (PVB) or single-shot intercostal nerve block (ICNB) is non-inferior to TEA regarding pain in patients undergoing VATS anatomic lung resection. Regarding QoR, PVB and ICNB are expected to be superior to TEA as scored by the global QoR-15 questionnaire. Signifying faster postoperative mobilisation, reduced morbidity and shorter hospitalisation, these locoregional unilateral techniques may therefore reduce health care costs and improve patient satisfaction.

### Objective

The main objective is to compare continuous PVB, single shot multi-level ICNB and TEA as analgesic techniques in order to provide safe, effective and efficient pain control and high quality of recovery after VATS lung resection. This study will provide evidence for the optimal analgesic technique after VATS anatomic lung resection to be implemented in an ERATS protocol.

### Study design

**Fig. 1:**
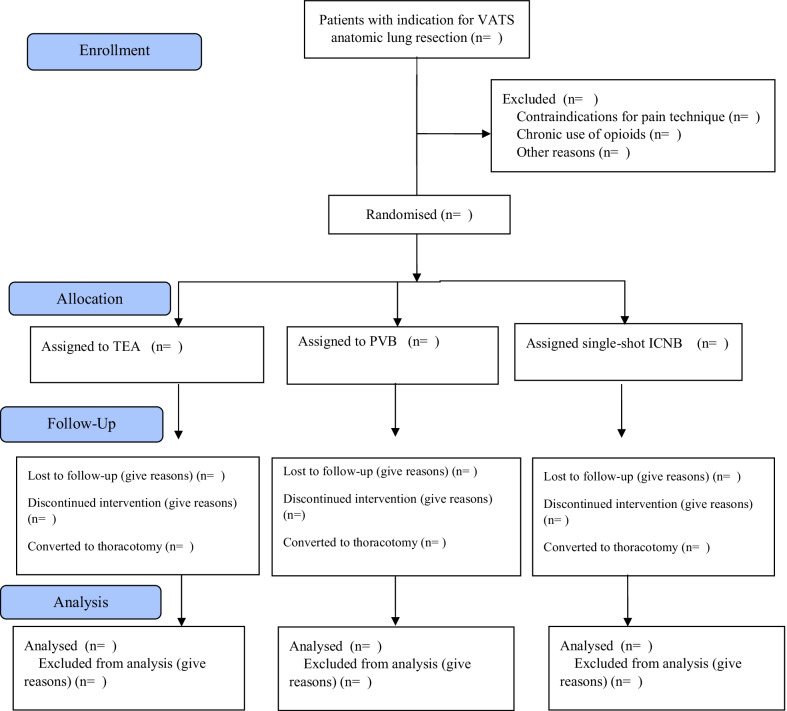
SPIRIT flow diagram

This is a multi-centre randomised three-arm trial comparing continuous PVB, single-shot ICNB and TEA in a 1:1:1 ratio in patients who will undergo a VATS anatomic lung resection. We use a non-inferiority design with respect to the outcome measure ‘pain’ and a concomitant superiority design regarding ‘quality of recovery’. The SPIRIT flow diagram is shown in Fig. 1.

### Randomisation and stratification

After informed consent, each local investigator with support of the principal investigator will enter patient data into a computerised database (Research Manager) and with an unchangeable computer generated number patients will be randomised (1:1:1) for one of the three analgesic strategies. Randomisation is done in blocks of 6, 9 and 12.

All participating hospitals use the international ERATS guideline [2] for the postoperative period, as a result of which only the randomised analgesic strategy will differ among patients. However, as local anaesthesiology protocols may slightly differ between hospitals regarding type of non-steroidal anti-inflammatory drugs (NSAID) use and general anaesthesia, and slight differences may exist in number of used ports/trocars during VATS, randomisation will be stratified by treatment centre.

### Blinding

As the analgesic strategies highly differ in nature (with or without percutaneous catheter) and/or postoperative care (mobility with or without prerequisites, urinary catheter placement), blinding for the randomised strategy is unfeasible.

### Inclusion criteria

Adult patients (> 18 years) referred for anatomic lung resection (pneumonectomy, (bi)lobectomy or segmentectomy for either benign or malignant disease) with the intention of performing it by VATS or robot-assisted thoracoscopic surgery (RATS) are eligible for the trial. Patients should be able to provide informed consent and fill out questionnaires in Dutch language.

### Exclusion criteria

Patients with contra-indications for TEA or PVB (infection at skin site, increased intracranial pressure, non-correctable coagulopathy, bridging indication for therapeutic anticoagulation (CHADS-VASc ≥ 8), sepsis and mechanical spine obstruction) or allergic reactions to local anaesthetics will be excluded. Patients chronically using opioids for reasons not related to the operation will be excluded from the study since postoperative baseline opioid requirement will be higher and TEA remains the preferred technique for these patients.

In case the lung surgeon estimates the operation to be performed through a thoracotomy instead of VATS/RATS the patient will be excluded.

### Primary outcomes

#### Pain

The numerical rating scale (NRS 0–10; 0 = no pain, 10 = worst imaginable pain) will be used to measure pain scores. A pain score ≥ 4 marks the clinical cut-off value for acceptable pain (NRS < 4) or not (NRS ≥ 4), since care-givers are triggered to provide additional pain medication in case of a NRS ≥ 4. The primary outcome measure for the ‘non-inferiority’ part of the study design is the proportion of NRS pain scores in rest ≥ 4, defined as the number of NRS ≥ 4 episodes divided by the total amount of NRS pain scores in rest obtained from POD 0 until POD 2. A minimum of 8 NRS pain scores will be collected (at the recovery room, on the ward the evening of the operation plus the following 2 postoperative days (PODs) in the morning, afternoon and evening). By using the proportion of NRS pain scores ≥ 4 we do not consider absolute pain scores (which lack power and are too subjective) but instead, rely on moments of adequate and inadequate pain control which are clinically more relevant.

#### Quality of recovery

The primary outcome for the ‘superiority’ part of the study design is QoR measured with the QoR-15 questionnaire on POD 1 and POD 2. Superiority in terms of QoR is defined as a clinically significantly higher score both at POD 1 and POD 2 or a higher score at least at POD 1 or POD 2 and the other score being at least equal (p < 0.05). The QoR-15 is a validated tool to measure overall quality of recovery and will provide a continuous variable with a minimum score of 0 and maximum score of 150, and contains the most relevant questions concerning 5 domains (emotional status, physical comfort, psychological support, physical independence and pain) of overall well-being and recovery after surgery [22–24].

### Secondary outcomes

1. Pain scores during rest and mobilisation at baseline, in the morning, afternoon and evening on POD 0–3 and at 2–3 weeks of follow-up;
2. Proportion of postoperative pain scores of NRS ≥ 4 during mobilisation at POD 0–3;
3. QoR-15 pre-operatively (baseline), at POD 0, POD 3 and at 2–3 weeks of follow-up;
4. Cumulative use of opioids and analgesics at POD 0–3 and the use of opioids at 2–3 weeks of follow-up; total opioid and non-opioid consumption as supplementary analgesic requirement during POD 0, POD 1, POD 2 and POD 3 as well as the use and dosage of opioid use at the follow-up period 2–3 weeks after the operation, when necessary the Opioid Oral Morphine Milligram Equivalent (MME) Conversion Factors table will be used;
5. Postoperative complications until 2–3 weeks of follow-up, according to the Clavien-Dindo classification;
6. Hospitalisation, defined as the total number of days in hospital after the surgical intervention (including readmissions within the first 30 postoperative days), the following standardised discharge criteria after surgery will be applied in all participating hospitals: normal intake of nutrition, independent mobility, absence of fever (< 38 °C), and no presence of chest tube;
7. Patient satisfaction regarding the analgesic technique given (5-point Likert scale: not at all satisfied, slightly satisfied, neutral, very satisfied and extremely satisfied);
8. Time to removal of chest drain in days;
9. Presence of urinary catheter in days;
10. Degree of mobility, measured each day during POD 0–3 [4-point scale: on the bed (1), to the chair (2), to the toilet (3), outside the patient’s hospital room(4)];
11. Cost-effectiveness of analgesic techniques from a health care perspective (see paragraph Cost-effectiveness analysis).

### Sample size calculation

A previous pilot study performed by our group [25], comparing TEA (n = 23) with subpleural continuous analgesia (n = 23), as a unilateral regional continuous analgesic technique, showed that 17.57% (SD 19.57) and 21.21% (SD 23.33) of the moments at which pain was measured postoperatively, patients had an NRS ≥ 4 at rest respectively. As research group, we consider the point estimators 17.57% and 21.21% as both low and find it clinically acceptable to set the non-inferiority upper margin regarding moments of pain at a difference of 17.5%, given the counterbalancing potential gain in QoR. Only if the upper limit of the 95% two-sided confidence interval (CI) of the difference in percentages of NRS measurements ≥ 4 between PVB and TEA remains below the 17.5% margin in the (modified) intention-to-treat analysis as well as the per-protocol analysis, we will reject the null hypothesis that PVB is worse than TEA in managing pain. For the distinct comparison between single shot ICBN versus TEA the same reasoning holds.

The estimated values used for the sample size regarding percentage of NRS moments in which NRS ≥ 4, are based on a single small pilot study of limited reliability. In addition, data from this pilot study showed a skewed distribution requiring non-parametric testing. Based on the Mann–Whitney U-test assuming that the actual distribution is normal and applying Dunnett’s correction to control the familywise error rate, while comparing two independent experimental groups with the same control group [26] we calculated that 64 patients were needed per group to achieve a power of 90% with a one-sided Type 1 error of 0.0135 to demonstrate non-inferiority of unilateral regional techniques. Based on an assumed 12.6% drop-out rate due to conversion of VATS/RATS to thoracotomy (Dutch Lung Cancer Audit data), we would need to include a total of 222 patients (74 per group).

We also calculated the needed sample size to demonstrate superiority of the unilateral regional techniques regarding QoR-15 (second primary outcome). QoR-15 is well reported in the literature and a difference of 8.0 points with a standard deviation of 18 points shows a clinically relevant difference [27]. Using this cut-off value for sample size calculation with a two-sample t-test, we initially need 125 patients per randomization group to achieve 90% power with a two-sided Type 1-error of 0.027 to control the family-wise error rate [26] in order to demonstrate superiority of the unilateral regional techniques. To account for possible non-normally distributed data and achieve 90% power with a Mann–Whitney U-test (with an asymptotic relative efficiency of 0.955 compared with the t-test) and assuming the abovementioned 12.6% drop-out rate, we aim to include 150 patients per group, or 450 patients in total. This sample size is sufficient to provide evidence for non-inferiority on pain as well, if our estimated values on percentage of NRS ≥ 4 are correct. A Data Safety Monitoring Board (DSMB) will be installed (1) to evaluate the point estimator and distribution of the control group (TEA) regarding the proportion of NRS ≥ 4 when 50% of observations in the control group are completed and (2) advise if further adjustment of the sample size is needed.

### Treatment of subjects and assessment of outcomes

At the preoperative outpatient clinic, during hospital admission, and at the postoperative outpatient clinic, patients will be asked to complete a number of questionnaires concerning the study end points. A detailed overview is shown in the schedule of assessments (Table 1).

**Table 1:** SPIRIT flow diagram: Schedule of assessments

Assessment	Indication for VATS anatomic resection after multidisciplinary meeting	Baseline (Pre-operative outpatient clinic consultation)	Day 0 (day of the operation)	POD* 1	POD 2	POD 3	Hospital discharge	Postoperative outpatient clinic consultation (2–3 weeks after operation)
Time point	t-1	t0	t1	t2	t3	t4	t5	t6
Assessment of eligibility	X	X**						
Written informed consent		X						
NRS pain score at rest		X						X
Morning				X	X	X		
Afternoon			X	X	X	X		
Evening			X	X	X	X		
NRS pain score during movement		X						X
Morning				X	X	X		
Afternoon			X	X	X	X		
Evening			X	X	X	X		
QoR-15 questionnaire		X	X	X	X	X	X	X
Dosage of used opioids and analgesics			X	X	X	X		X
Patient satisfaction			X	X	X	X		
Postoperative complications							X	X
Patient mobility			X	X	X	X		
Hospitalisation***								X
iMTA—iMCQ		X						X
iMTA—iPCQ		X						X

*POD: postoperative day

**Assessment of eligibility can take place during a multidisciplinary meeting or can take place if the patient is referred to the surgeon for anatomic lung resection without a multidisciplinary meeting beforehand

***Readmission within 30 days after surgery

### Preoperative analgesics

All patients will receive paracetamol (acetaminophen) 1000 mg. In addition, NSAID will be given according to in house protocol unless contra-indications exist. All preoperative analgesics administered will be registered in the case report form (CRF).

### General anaesthesia

For induction and maintenance of anaesthesia in house protocols will be used with the exception of lidocaine or esketamine which will not be administered during general anaesthesia. All patients will receive 8 mg dexamethasone to reduce additional postoperative opioid requirements and aid in the prevention of postoperative nausea and vomiting. In addition, a 5HT3 receptor antagonist will be administered and additional anti-emetics based on risk factors and local protocols.

### Intervention and usual care

#### Usual care (Group 1: TEA)

The epidural catheter will be placed in the awake patient after local anaesthesia of the skin. After correct placement of the epidural catheter, a local anaesthetic (ropivacaine, levobupivacaine or bupivacaine) will be started and, according to in house protocols, an opioid will be added to the epidural solution. In the nursing ward, patients are allowed to mobilise under supervision when the motor function and sensibility of the extremities allows it. A provisional stop of the administration of the epidural infusion is planned after 48 h (on the second postoperative day). In case NRS pain scores are ≥ 4 despite additional pain medication, the TEA will stay in place and the epidural infusion is resumed after a bolus of 5 mL of the epidural infusion. Subsequently, NRS pain scores will be assessed daily until pain management is sufficient and the TEA can be withdrawn with a maximum of 4 days. If rescue attempts to the epidural anaesthesia fail to improve pain scores, opioids may be withdrawn from the epidural solution and oral or intravenous opioids will be supplied.

### Interventions

#### Group 2: continuous regional PVB

After induction of general anaesthesia the patient is positioned in lateral decubitus and the relevant landmarks are identified and marked (midline of the vertebral column and 2–3 cm next to the midline where palpation does not meet the transverse process). The PVB catheter is placed under general anaesthesia at the beginning of the VATS procedure under direct thoracoscopic vision. If placement cannot be achieved at the beginning of the operation as a result of poor thoracoscopic vision (e.g. adhesions) the catheter will be placed at a later stage or at the end of surgery. It is strongly preferred to place the PVB at the beginning of the VATS procedure to benefit from the advantages of administering local anaesthetics during general anaesthesia.

After introduction to the thorax and manipulation of the lung anteriorly, the sympathetic chain running parallel to the vertebral column is identified with thoracoscopy. The level of the PVB catheter placement is chosen at the intercostal space of the largest incision (mostly thoracic level 4 or 5). Under direct thoracoscopic vision, the surgeon inserts a Touhy needle at the before mentioned marked location. After feeling a “fascial pop” penetrating the intercostal ligament, loss of resistance is felt when entering the subpleural space. At the same time, the tip of the needle is observed beneath the pleural surface thoracoscopically. Injection of about 2 mL ropivacaine 7.5 mg/mL will create subpleural hydrodissection to reach the adequate paravertebral plane for placement of the catheter. The PVB catheter is subsequently placed under direct thoracoscopic vision and left next to the sympathetic chain in the paravertebral space. Next, a bolus of ropivacaine (total amount 20 mL including the given amount for hydrodissection) is given through the catheter. The catheter will be fixed to the skin.

Postoperatively, a ropivacaine 2 mg/mL pump for continuous infusion is given with an infusion rate of 8–14 ml/h. In case of insufficient pain control (NRS ≥ 4) a bolus of 4–5 mL is given (in case a patient controlled (epidural) anaesthesia pump is available with a lockout of 20 min). No opioid additives or opioids will be administered through the paravertebral catheter. A provisional stop of the administration of local anaesthetics is planned on the second postoperative day after which removal is considered based on the effect of pain intensity, with a maximum of 4 days, comparable to the TEA group. No mobility restrictions are instructed in this group.

#### Group 3: single shot ICNB

At the end of the surgery a single shot ICNB will be placed at 9 levels (thoracic level 2 to 10) with 2-3 mL local anaesthetics per intercostal space under direct thoracoscopic vision. The injection site will be chosen just lateral from the sympathetic trunk. This group will have no analgesic catheters for continuous analgesia. No mobility restrictions are instructed in this group.

If catheter placement during TEA is unsuccessful, a continuous PVB will be given during the procedure, and, if catheter placement during PVB is unsuccessful, a single shot multilevel ICNB will be used. Non-inferiority will be analysed based on intention-to-treat, as well as per-protocol analysis.

### Escape medication at the recovery room

If postoperatively at the recovery room the patient experiences inadequate pain control (NRS ≥ 4) and a bolus of local anaesthetic via the epidural or paravertebral catheter (if present) is insufficient, intravenous morphine will be given until a maximum dose specified by the attending anaesthesiologist. If still insufficient pain control is achieved, additional clonidine 1 μg/kg or esketamine (depending on patient’s hemodynamics and local protocol) is injected intravenously in order to obtain adequate pain control (NRS < 4). If the above regime does not result in adequate pain control additional interventions will be administered at the discretion of the attending anaesthesiologist. All analgesic medications and interventions given will be documented and registered for the purpose of the study.

### Postoperative medication at the nursing ward

The following medication will be provided to each patient: paracetamol (acetaminophen) 4 times a day 1000 mg, NSAID according to in house protocols and oxycodon 6 times a day 5 mg if needed. If this regime does not provide adequate pain control, additional opioids will be provided either orally (slow release) or intravenously by patient controlled analgesia. The latter can either be initiated in the recovery room or on the nursing ward.

### Recruitment and consent

**Fig. 2:**
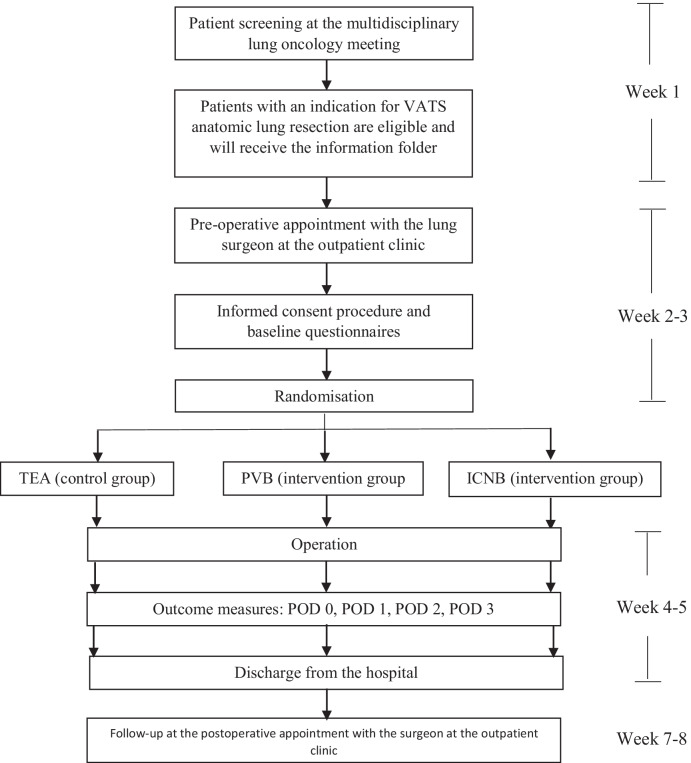
SPIRIT diagram: timeline overview of recruitment and consent

Consecutive patients who fulfil the inclusion criteria will be noticed during the local multidisciplinary lung oncology meeting or at the outpatient clinic. They will receive written or digital study information. At the preoperative outpatient clinic consultation, patients will have the possibility to ask questions and have additional explanation. Written informed consent must be obtained before inclusion and randomisation, that will be done by a computerised program, using Research Manager Software. Subsequently, the patient will receive the complete information about the randomised anaesthesia technique by the anaesthesiologist and will be scheduled for surgery. Patients unable or refusing to provide informed consent will be treated according to current clinical guidelines. For the SPIRIT diagram of recruitment and consent see Fig. 2.

### Quality assurance

All participating centres will have a detailed hands-on training on how to perform PVB and ICNB. This training will be held by the researchers for all participating surgeons and anaesthesiologists. During the first PVB and ICNB procedures within the study, trained personnel will attend the participating surgeon/anaesthesiologist at location until sufficient experience is reached. All VATS procedures are recorded and video’s will be controlled for distance monitoring and if there are any problems, extra visits to the participating hospitals will be planned. For TEA (usual care), all participating centres should adhere to local anaesthesia guidelines. Training by this protocol will guarantee standard execution of the interventions and high quality performance of the three different analgesic techniques.

### Statistical analysis

#### Primary outcome

The proportion of postoperative pain scores of NRS ≥ 4 at rest will be presented as mean or median percentages with 95% confidence intervals (95-CI) depending on the distribution and the QoR-15 questionnaire scores (maximum 150 points) will be presented as means with standard deviation or medians with interquartile range depending on distribution. Consequently, comparisons for both primary outcomes will be made with student’s t-test or Mann–Whitney U-test.

When both intervention groups turn out to be non-inferior to TEA regarding pain, a comparison on clinical outcome and cost-effectiveness will be done between the different intervention groups.

#### Secondary study parameters

The three randomisation groups will also be compared with respect to the following secondary outcome measures. Continuous variables will be described by means with SD or medians with IQR depending on distribution of data. Comparisons will be made using the student’s t-test or Mann Whitney U test respectively. In case of proportions (with 95-CI), comparisons are tested by the chi-squared test.

1. Pain scores during rest and mobilising at baseline, the morning, afternoon and evening at POD 0–3 and at 2–3 weeks follow-up; NRS on a scale of 0–10, will be presented as means with standard deviation or median with interquartile range depending on distribution.
2. Proportion of postoperative pain scores of NRS ≥ 4 during mobilisation at POD 0–3; will be presented as percentages with 95% confidence interval (95-CI).
3. QoR-15 pre-operatively as baseline score, at POD 0, POD 3 and at 2–3 weeks follow-up; questionnaire scores (maximum 150 points) will be presented as means with standard deviation or median with interquartile range depending on distribution.
4. Cumulative use of systemic opioids at POD 0–3; will be presented in the measure of milligrams (mg) using means with standard deviation or median with interquartile range depending on distribution.
5. Postoperative complications until 2–3 weeks follow-up, according the Clavien-Dindo classification (Grade I–V); proportions with minor (grade I–II) and major (grade III–V) morbidity.
6. Hospitalisation, defined as the total number of days in hospital after the surgical intervention (including readmissions within the first 30 days postoperatively), will be presented as means with standard deviation or median with interquartile range depending on distribution.
7. Patient satisfaction (5-point Likert scale: not at all satisfied, slightly satisfied, neutral, very satisfied and extremely satisfied); evaluated as proportion of patients per category for time points t1, t2, t3 and t4 (see Table 1).
8. Time to removal of thorax drain in days; will be reported as means with standard deviation or median with interquartile range depending on distribution and presented as Kaplan–Meier curves.
9. Time to removal of urinary catheter in days; will be reported as means with standard deviation or median with interquartile range depending on distribution and presented as Kaplan–Meier curves.
10. Degree of mobility (on the bed, to the chair, to the toilet, outside patient’s hospital room); evaluated as proportion of patients per category for time points t1, t2, t3 and t4.

### Cost-effectiveness analysis

The economic evaluation of PVB and ICNB against TEA will be performed as cost-effectiveness analysis from a health care and societal perspective in this heavily affected and mostly already specialist dependent patient population. The primary outcome for cost-effectiveness analysis is the costs per total QoR score as continuous outcome measure. Additionally, we will analyse the costs per patient with adequate pain control (defined as NRS ≤ 4). The time horizon is restricted to a follow-up of 30 days after surgery. Incremental cost-effectiveness ratios are calculated, reflecting the extra costs per score of QoR and per patient with adequate pain control. Sensitivity analyses will be performed to account for sampling variability (following bias corrected and accelerated non-parametric bootstrapping), for plausible ranges in unit costs of surgical and anaesthesiologic treatment, and for (differential) discount rates of costs and effects. Subgroup analyses will be performed for patients treated by uniportal or multiportal VATS in order to tentatively assess differences in health care efficiency. In case all analgesic strategies turn out clinically equivalent, the study will be performed as a cost-minimization analysis.

The cost analysis evaluation will include direct medical costs, out-of-pocket expenses, and indirect non-medical costs of production loss. Volume data will be gathered with clinical report forms, available hospital information systems, and the iMTA Medical Consumption Questionnaire (iMCQ) and iMTA Productivity Cost Questionnaire (iPCQ) adjusted to the study setting (to be completed by patients at baseline T0 and 30 days after surgery T6. Micro-costing (general anaesthesia, surgical and anaesthesiologic equipment, procedure duration, involved personnel, and overhead) in participating centres will be done to estimate real unit costs. The friction costs method will be applied to derive the costs of lost productivity. After price-indexing all costs will be expressed in Euros.

The budget impact analysis (BIA) focusing on the budget of medical specialist care will be done with a planning horizon of 4 calendar years, addressing the governmental, insurer and provider perspectives. Alternative impact assessments will be made based on (1) real unit costs and (2) reimbursements. Different national implementation scenarios of unilateral regional analgesic techniques replacing TEA will be forecasted. Budget impacts will be expressed in millions of Euros.

### Data safety and data management

After randomisation patients will be assigned a study number and anonymous data will be registered. Data is registered and stored in Research Manager Software. Research Manager software is certified by the ‘Information Security Management System 27001’. The key to the code is safeguarded by the principal investigator.

Local data management will be done by Integraal Kankercentrum Nederland (IKNL), having extensive experience with management of local data collection. Collection, storage and analysis of data will be done according to the OPtriAL data management plan.

### Adverse events and harms monitoring

According to the risk classification, the OPtriAL has negligible risk for the study subjects. All patients, regardless of the randomisation group, will have monitoring of vital parameters and direct presence of experts in the field if required. Moreover, there will be no new medicinal intervention and all doses of applied anaesthetics are already used in daily practice.

All adverse events per participant will be recorded until the end of the study and followed until they have abated, or until a stable situation has been reached. Depending on the event, follow up may require additional tests or medical procedures as indicated, and/or referral to the general physician or a medical specialist.

### Data safety monitoring board and interim analysis

The uncertainty in the estimated values and distribution that have been used in the sample size calculations for our first primary outcome measure (proportion of NRS measurements ≥ 4) require a more solid estimation, based on a larger sample size than achieved in our pilot study. Inclusion of 50% of the number of TEA patients based on the sample size calculation to demonstrate superiority of QoR-15 (second primary outcome for superiority) will define the moment for a blinded interim analysis. At this point (inclusion of 75 patients in the TEA group), an interim analysis by our data safety monitoring board (DSMB) will establish a more precise estimate of the proportion and distribution of NRS measurements ≥ 4 for the control group (TEA). Normal or skewed distribution of the study groups will determine the use of parametric or non-parametric statistical tests, respectively. With the new estimators, the already defined clinically relevant contrasts of 17.5% for the NRS ≥ 4, 90% target power and corrected Type-1 errors of 0.0135, the DSMB will recalculate the sample size with the correct statistical tests and advise the research group whether the OPtriAL study should continue or stop the inclusion of patients after the calculated sample size of 450 patients in total.

During the blinded evaluation by our DSMB, our patient recruitment will continue in order to not disrupt the complex logistics that have been set up in the multicentre setting of this national trial.

### Monitoring and auditing

Monitoring of the participating centres will be done by IKNL according to the OPtriAL monitoring plan. The sponsor location will be monitored by CTCM (Clinical Trial Centre Maastricht).

All centres will be visited three times during the execution of the inclusion of study participants until finalisation of the study inclusion after a minimal period of 2 years. Remote visits are planned if needed depending on inclusion rate and queries in data management. Monitoring will take place with specific attention to informed consent, data monitoring and completeness of case report form.

## Discussion

Large variety exists among surgeons and anaesthesiologist in applying either TEA or continuous or single-shot loco-regional analgesic techniques or even only systemic analgesia in the setting of VATS for anatomical lung resection. This variety may mainly be explained by the fact that no large randomised comparisons have been made between TEA and continuous or single-shot loco-regional analgesia in combination with a multimodal analgesic regime. Although TEA has a long history and anaesthesiologists have experience with this technique, associated patient immobilisation and indwelling urinary catheter usage may lead to prolonged hospital admission [2]. Reducing the length of hospital admission and morbidity, as well as eliminating the need of awake placement of TEA, may result in increased patient satisfaction and a more cost-effective strategy.

The results of the proposed study, which is being performed in multicentre setting within the Netherlands and Belgium, may have direct impact on national and international guidelines to optimize perioperative care for VATS anatomic lung resection. Besides determining the most effective and efficient analgesic technique, taking into account patient’s satisfaction, it will also determine the most cost-effective pain strategy and will eventually reduce variability in postoperative pain management.

## Supplementary information

**Additional file 1.** Supplemental material.

## Data Availability

The datasets and/or analysed data will be open access and available from the corresponding author on reasonable request. For more information please see our current data management plan in the Additional file 1.

## Abbreviations

CRF: Case report form
ERATS: Enhanced recovery after thoracic surgery
ICNB: InterCostal nerve block
NRS: Numerical rating scale
POD: PostOperative day
POS: PreOperative screening
PVB: ParaVertebral block
QoR-15: Quality of recovery-15 questionnaire
RATS: Robot assisted thoracoscopic surgery
VATS: Video assisted thoracic surgery
TEA: Thoracic epidural anaesthesia
