# Supplementary material for: Optimal postoperative pain management after VATS lung resection by thoracic epidural analgesia, continuous paravertebral block or single-shot intercostal nerve block (OPtriAL): study protocol of a three-arm multicentre randomised controlled trial
Source: BMC Surg. 2022 Sep 4;22:330. doi: 10.1186/s12893-022-01765-y (PMC9441091; doi:10.1186/s12893-022-01765-y)
Supplement: Supplementary file 1 — Additional file 1. Supplemental material. [file 12893_2022_1765_MOESM1_ESM.pdf]

Supplemental material

Pertaining to the following manuscript:

Optimal postoperative Pain management After VATS Lung resection by thoracic epidural analgesia, continuous paravertebral block or single-shot intercostal nerve block (OPtriAL): study protocol of a three-arm multicentre randomised controlled trial

L.N. Spaans[1,2], M.G.W. Dijkgraaf [2,3], P. Meijer[4], J. Mourisse[5], R.A. Bouwman[6], A.F.T.M. Verhagen[7], F.J.C. van den Broek[1]*, OPtriAL study group

* Corresponding author

[1] Department of Surgery, Máxima Medical Center, Veldhoven, The Netherlands.

[2] University of Amsterdam, Amsterdam, The Netherlands.

[3] Department of Epidemiology and Data Science, Amsterdam University Medical Centers, University of Amsterdam, Amsterdam, The Netherlands.

[4] Department of Anesthesiology, Máxima Medical Center, Veldhoven, The Netherlands.

[5] Department of Anesthesiology, Radboud University Medical Center, Nijmegen, The Netherlands.

[6] Department of Anaesthesiology, Catharina Hospital, Eindhoven, The Netherlands

[7] Department of Cardiothoracic Surgery, Radboud University Medical Center, Nijmegen, The Netherlands.

Inhoud

Data management plan (DMP)* 3
Patient information folder* 8
List of participating centers 18

*Translated from Dutch

Data management plan (DMP)*
*Translated from Dutch

1. Features of the project and data collection

1.1 Contact details project leader

F.J.C. van den Broek

Máxima Medical Center, Veldhoven, surgeon, Department of Surgery

De Run 4600, 5504 DB, Veldhoven

Tel: 040-888-4110, E-mail: frankvanden.broek@mmc.nl

1.2 I have prepared my DMP in collaboration with a data management expert. State name, position, organization/department, telephone number, e-mail address.

• The expert is from outside my department/institute

R.A. Scholte, Head of data management

Academic Medical Center Amsterdam, Clinical Research Unit, Head of Data Management

Meibergdreef 9, 1105 AZ, Amsterdam

Tel: 020-667649, E-mail: r.a.scholte@amc.nl

1.3 When collecting the data for my project, I proceed as follows:

• Use existing data (name)

• Generate new data

(Existing) clinical data from the patient electronic platform is used: age, gender, pain medication use, tumor location, tumor classification and reports (OR/pathology/anesthesia).

New data is generated by means of questionnaires: pain scores, QoR-15, patient satisfaction and mobility after surgery (objectified by nurse or ward physician).

1.4 In my research I use:

• Quantitative data only

The questionnaires regarding pain, quality of recovery, opioid use, mobilization and co-morbidities will be expressed as quantitative data. In addition, the new data from the patient preference study will be structured in quantitative data.

1.5 I am going to reuse existing data and/or link his/their data

• Yes, I have permission to use his/their data.

For the use of clinical patient electronic platform data, permission is given by both the patient (informed consent) and the primary practitioner (research contract) for use.

1.6 When collecting new data, I work together with other parties

• Yes, I collect new data together with other researchers, research groups (multicenter research).

Local researchers and data managers process the data via the electronic case report form (eCRF) in research manager. Associated datasets are encoded as SPSS files.

The agreements about the accessibility, reusability, exchangeability and verifiability of the (new) data set and about ownership or co-producership of data will be established in writing with each participating center and principal investigator in a research contract.

1.7 I carry out the project in a consortium with two or more partners. Clear agreements have been made within the consortium about data management and intellectual property.

• Yes, clear agreements have been made with regard to data management and intellectual property through a consortium agreement.

1.8 I can estimate the size of the data file, namely the number of participants or subjects ("n=") of the data collection and the size in giga-/terabytes.

• Yes

Sample size calculation: n= 450 patients, <1 GB of data.

1.9 I will make the following end products of the project available for follow-up research and verification. (explain briefly)

• (different versions of) edited data

• Documentation about the data

• Documentation about the research process, including data from all involved

Edited data on which the reports for scientific articles are based. The raw data will be translated into different variables and made available in coded form. The codebook will indicate which code belongs to which variable. We also report the method of data collection, which questionnaire is administered at what time, how we score the questionnaires and how the clinical data is coded. Due to privacy reasons, the raw data will not be available for further research.

1.10 During the project I have sufficient storage locations and capacity and I have a backup of the data available. (Give a brief explanation)

• Yes, I use the standard facilities of my institution for the storage and backup of my data.

Clinical data collection and storage is done with Research Manager. Local storage of the data after the data collection phase (processing and analysis), protocols, contracts, documents and data processing software on the secure disk of the Máxima Medical Center.

2. Legislation and regulations (including privacy)

2.1 I am going to conduct human research and I declare that I am aware of and comply with the laws and regulations regarding privacy-sensitive data

• Personal Data Protection Act and the resulting code of conduct for Health Research. I register my project with the Dutch Data Protection Authority

• Quality Assurance of Human-Based Research

• Medical-Scientific Research with Humans Act (WMO). I submit my project for review to a Medical Ethics Review Committee

• Medical Treatment Agreement Act

2.2 I am going to conduct human research and I have arranged that I obtain data with (a form of) permission from the participants.

• Yes, state the form of consent.

• Yes, the consent form allows data reuse

Research contract signed per study site.

Research statement signed by the local department heads of the study site.

Written informed consent for participants giving consent to participate in this study (randomisation, treatment, data analysis, questionnaire completion, completion of follow-up) and to reuse data and approach patients for follow-up research.

2.3 I am going to conduct human research and I am going to anonymize or pseudonymize privacy-sensitive data.

• Yes, I will have the data pseudonymised.

Patients are given a code (example OPtriAL-001), the key code list remains in the center with the local principal investigator and is stored separately for possible later checking. The name and date of birth is not used in the analysis.

2.4 I adhere to the privacy regulations of the organization to which I am affiliated

• Yes

3. Make data discoverable

3.1 The data collection from my project can be found for further research. (Note: This is a key information that you must pass on to ZonMw at the end of your project.)

• Yes, via the search engine of the archive (repository) in which it is stored (name)

We will use an external archive, namely DANS.

3.2 For the description of the data collection I use a metadata standard.

• No, I have not yet made a choice for a metadata standard.

The description of the data collection will be done on the basis of validated questionnaires. In the case of patient (clinical) data, this variable will be described using a codebook.

3.3 I will use a Persistent Identifier (PI) to permanently reference the data file. (Note: This is a key information that you must pass on to ZonMw at the end of your project.)

• Yes, the doi code

4. Making data accessible

4.1 After the end of the project, the data will be accessible for verification and follow-up research.

• Yes, after an embargo period (explain)

Data will become available for non-commercial scientific research (open access) after a period of 12 months after the last data collection.

4.2 At the end of the project, the database will be made publicly available, without additional conditions (open access).

• No, I attach conditions to access to the data file (restricted access) (explain)

In collaboration with a lawyer from MMC, we have applied a set of general terms and conditions for reuse. These can be found in the research contract with each study site.

4.3 I have terms of use available that explain the conditions for accessing my database after the project is finished (provide a link or Persistent Identifier). (Note: This is key information that you must pass on to ZonMw at the end of your project).

• No, my institute will draw up the terms of use in collaboration with a lawyer

4.4 In the conditions that I set for the use of my data (restricted access), I have in any case included the marked points below.

• Conditions related to data security

• Agreements on methodology

• Whether the dataset may be linked to another dataset (privacy)

• Sharing data for commercial purposes. In doing so, I take into account the provisions of state aid law.

• Collaboration in using the dataset, including agreements on publications, authorships

• The way the dataset is made available

• The period of permission to use the dataset

• A steering group, program committee or project leader will decide on the approval of data requests

• Consent of the participants allows for further research with the dataset

Data may not be used for commercial purposes.

5. Making data interoperable

5.1 I choose a data format so that my data is readable by other researchers and their computers ('machine actionable').

• Yes, name

SPSS file

5.2 I choose a metadata standard so that my data can be linked to other data. (Note: this is a key information that you must pass on to ZonMw at the end of your project).

• No

There is no common metadata standard in our field. We use validated questionnaires. Such questionnaires are widely used in our field and for that reason the data will be made interoperable in this way. In the case of patients (clinical data), the data will be described using a code book.

5.3 I am going to conduct research related to people and have taken into account the reuse of the data and any link with other data files when protecting privacy.

• Yes, the participants have given permission for the data to be reused and the data has been pseudonymised

Written informed consent

6. Making data reusable and storing it sustainably

6.1 I ensure good quality and documentation of the data so that other researchers can interpret and use it (replication package).

• I document the research process (explain)

• I perform quality checks on the data so that they are complete, correct and consistent (explain)

Data collection is done through Research Manager. At each follow-up moment, an eCRF is entered by the IKNL data manager. Checking for completeness is done by the doctor-researcher working on the project.

External quality monitoring is done by IKNL. All centers are visited at the beginning and end of the study and, moreover, a visit is made in between. In the course of the study, extra monitor visits will follow at centers where there is a lot or little inclusion, many queries in data management or other reasons that lead the PI to plan additional monitor visits. At the end of the study, all centers will be visited by the researcher physician.

6.2 I have selection criteria to determine at the end of the project which part of the data should be kept.

• Yes

All data will be kept for reproducibility. Depending on the end products in terms of reports and articles, the processed and used data is still stored per product.

6.3 At the end of the project, after selecting the data, I can estimate the size of the data file (in Gb/Tb) that I will be storing/archiving for the long term.

• Yes

Presumably <1 GB gigabyte

6.4 I make sure that at the end of the project I have made a choice for an archive or repository for sustainable long-term archiving (certified) of my data file. (Note: This is a key information that you must pass on to ZonMw at the end of your project.)

• Yes, the archive has a data seal of approval (name the archive)

DANS

6.5 After the project, I will use the recommended retention period of at least 10 years for my data

• Yes (mention number of years)

15 years according to the Medical Research with Humans Act (WMO) and Good Clinical Practice (GCP) guidelines.

6.6 The costs for data management during the project and the preparation of archiving may be included in the project budget. The costs are:

The costs have been included in the budget to ZonMw. The costs for local data management based on a multicenter trial with at least 10 participating centers and taking into account the protocol and CRF is (an estimate of) 128,458.05 euros. Central data management, drafting a codebook and a data file in SPSS are tasks that are performed by the physician-researcher.

6.7 The costs of (preparing the data for) archiving are covered.

• Yes (explain)

The costs for storing the data from questionnaires are borne by IKNL. The costs for storing the clinical data in research manager are included in the license that the Máxima Medical Center has. The costs for storing local documents and analysis software are borne by Máxima Medical Center.

Patient information folder*

*Translated from Dutch

Subject information for participation to medical scientific research

Optimal Postoperative Pain Management After Lung Surgery (OPtriAL): A Multicenter Randomized Clinical Trial

Introduction

Dear Sir / Madam,

With this information letter we would like to ask you if you would like to participate in medical research. Participation is voluntary. You are receiving this letter because a lung abnormality has been found in you and you will therefore soon be undergoing lung surgery where part of the lung will be removed.

Here you can read about the research involved, what it means for you, and what the advantages and disadvantages are. It's a lot of information. Would you like to read through the information and decide if you want to participate? If you want to participate, you can fill in the form that you can find in Appendix B.

Ask your questions

You can make your decision with the information you will find in this information letter. In addition, we recommend that you do this:

- Ask questions to the researcher who gives you this information.

- Talk to your partner, family or friends about this study.

- Ask questions to the independent expert, Dr. Mark van den Baar.

- On our website you will find an understandable patient information video: www.optrial.com

- Read the information at www.rijksoverheid.nl/mensenonderzoek

1. General information

The Máxima Medical Center has set up this research. Below we refer to the Máxima Medical Center as the 'sponsor'. Researchers, this can be lung surgeons, anaesthesiologists, doctors in training, medical researchers or nurses, carry out the research in various hospitals in the Netherlands and Belgium. This research was made possible and funded by ZonMw.

450 subjects are needed for this study.

The Utrecht Medical Ethics Review Committee has approved this study. General information about the assessment of research can be found on the website of the national government: www.rijksoverheid.nl/mensenonderzoek

2. What is the purpose of the research?

The aim of this study is to investigate the best technique for treating pain after minimally invasive lung surgery in which part of the lung is removed. With this operation it is important that good pain relief is administered so that you can get out of bed as quickly as possible, breathe well and cough with as little pain as possible.

The intention is to publish the results of this research in a medical journal. We also want to use the results to draw up a national guideline. With this we hope that in the future all patients who undergo minimally invasive lung surgery in which part of the lung is removed will receive good pain relief and experience a better recovery.

3. What is the background of the research?

Patients who undergo minimally invasive lung surgery have to deal with pain after the operation. There are several techniques to treat this pain. These are 1) an epidural, 2) continuous local pain relief and 3) single-shot local pain relief. The epidural is the most commonly used, but the other techniques are already being used throughout the Netherlands. It is not yet known which technique is the best.

The epidural is the most commonly used because it seems to work best for the pain and doctors have the most experience with this. A bladder catheter is required and for safety reasons you may only get out of bed under supervision. It is good for your recovery if you get out of bed soon after the operation and start moving. With continuous and single-shot local pain relief, the nerves that respond to pain are switched off as much as possible by injecting an anesthetic around a nerve. In the case of continuous local pain relief, this is done through a catheter that is placed near the nerve. With these techniques you do not need a bladder catheter and you can get out of bed independently. This may allow you to go home earlier. Recent research shows that this local painkiller can cause a little more pain. Despite this, on average 8 out of 10 people were satisfied with these techniques.

4. How is the investigation progressing?

How long does the investigation take place?

If you participate, the study will take you about 2 months in total.

Step 1: are you suitable to participate?

We first want to know if you are suitable to participate.

This is first determined by the surgeon with whom you have a conversation before the operation. The surgeon will ask whether you often have pain (chronic pain) and whether you use strong painkillers for this. If you take these medicines on a daily basis, you cannot participate in this study. If you indicate that you are allergic or do not qualify for one of the pain techniques that are being studied due to personal factors, you will not be able to participate in this study.

Step 2: choice of pain treatment during surgery

During your visit to the surgeon before the operation, during which an explanation is given about the study and the operation, it will be determined whether you can participate. If you are eligible and want to participate, you sign for consent together with the surgeon (so-called informed consent). Subsequently, it is determined by randomization which pain technique you will receive during the operation. A third of the subjects receives an epidural, a third receives continuous local pain relief and a third receives one-time local pain relief. The randomization determines which treatment you will receive, so you cannot choose yourself.

Group 1: Epidural

The epidural is performed by an experienced anesthesiologist before surgery and before anesthesia. You are awake during this operation. The skin on the back is anesthetized with a prick and then a thin catheter is inserted into your back (between the vertebrae) by using a hollow needle. The needle is removed and the catheter is taped. A pump is connected to this that ensures continuous administration of pain relief. There is a chance that the catheter placement is not successful, which means that a different method of pain relief is required. The area from your chest down to the thighs can be numbed, so that you can experience muscle weakness in the legs. For this reason, you will be given a bladder catheter and you will not be allowed to get out of bed unaccompanied. The tube is usually removed after 3 days, after which you will receive pain relief, if necessary, as tablets or via an IV in your arm.

Group 2: Continuous local pain relief

This technique is performed by an experienced lung surgeon at the beginning of the operation while you are under anesthesia. Continuous local pain relief means that constant pain relief is given by means of a thin catheter that is inserted under general anesthesia through the back next to the vertebrae. This catheter is located at the location of the nerves of the surgical site. After surgery, a pump is connected to the catheter for continuous administration of pain relief in the surgical area. The catheter is usually removed after 3 days, after which you will receive pain relief, if necessary, as tablets or via an IV in your arm.

Group 3: One-time local pain relief

This technique is performed by an experienced lung surgeon at the end of the operation while you are under anesthesia. With a single-shot local pain relief, an analgesic medicine is administered once you are under general anesthesia at the site of the nerves of the surgical site with a syringe. This pain relief works for about 6-12 hours and afterwards you will receive pain relief as tablets or through the IV in your arm.

Groups 2 and 3 do not require a bladder catheter to be placed and there are no restrictions on getting out of bed. If the pain symptoms are unbearable with the local pain relief technique despite extra pain relief that you have received, an epidural can still be given in consultation with you to offer the maximum pain relief.

Step 3: questionnaires before and during the admission

It is not necessary for you to come to the hospital for the examination. We use the standard visits before surgery, during hospitalization and the first outpatient visit after surgery to administer questionnaires. Among other things, you will be asked how you experience the pain and how satisfied you are with the treatment of the pain. Pain scores are recorded three times a day. A questionnaire will also be administered about the quality of your recovery and about your use of care and ability to work.

Below is an overview of the times when we conduct questionnaires and the time investment that is required of you.

Measuring moment	Standard visit	What is asked of you?
1	First outpatient appointment with the surgeon	35 min
2	After surgery while in hospital	5 min
3	1 day after surgery *	5 min
4	2 days after surgery *	5 min
5	3 days after surgery *	5 min
6	During the first check-up outpatient visit with the surgeon	35 min

* You fill in these questions in a diary together with the nurse on the ward

What is different from normal care?

In your hospital, the standard pain relief for lung surgery is an epidural.

If you decide not to participate in the study, you will be treated according to this standard pain relief technique.

If you decide to participate in the study, you will be randomly assigned to one of the three groups, as described in section 4. You will also receive questionnaires regarding pain and quality of recovery.

5. What agreements do we make with you?

We want the investigation to go well. That is why we make the following agreements with you:

• You come to every appointment.

• You contact the researcher in these situations:

o You are sick and admitted to the hospital or treated at home.

o You suddenly have problems with your health.

o You no longer wish to participate in the study.

o Your phone number, address or email address changes.

There are no restrictions on eating, drinking or other activities of your daily life.

6. What side effects, adverse effects or inconveniences may you experience?

Despite all due care, complications cannot always be prevented. The techniques used in this study to minimize pain are often used. It is very rare for something to go wrong.

<u>Epidural</u>

The following side effects/adverse effects may occur with this technique:

• pain during sting (the skin is first numbed with a short-term painful sting),

• low blood pressure (which can make you a little light-headed),

• difficulty urinating (requiring a urinary catheter),

• inflammation of the area where the back is punctured,

• bleeding at the puncture site,

• nerve damage (very rare).

Continuous local pain relief

The following complications can occur with this technique:

• bleeding or inflammation of the puncture area.

This is very rare. If it occurs, you can sometimes have temporary pain symptoms.

Single-shot local pain relief

The following complications can occur with this technique:

• bleeding or inflammation of the puncture area.

This is very rare. If it occurs, you can sometimes have temporary pain symptoms.

7. What are the advantages and disadvantages of participating in the study?

You may not benefit from participating in this study yourself. Your participation can contribute to more knowledge about the optimal pain treatment after minimally invasive surgery of the lung where part of the lung is removed. With this information we can improve recovery after surgery and pain relief in the future.

It is important that you carefully consider the possible advantages and disadvantages before you decide to participate.

Possible disadvantages of participating in the study may include:

- that randomization determines the method of pain relief

- that you spend extra time filling out questionnaires

Don't want to participate?

You decide whether you want to participate in the study. Don't want to participate? Then you will be treated in your hospital according to the standard pain relief method.

8. When does the research end?

The study will stop for you if:

• The first outpatient check-up after the operation is over

• You choose to stop

• Your attending physician, the government or the reviewing medical-ethical review committee, decides to stop the study.

What happens if you stop the study?

If you do participate, you can always change your mind and stop anyway, even during the study. You don't have to say why you're stopping. You must report this to the researcher immediately.

The data collected up to that point will be used for the research.

9. What happens after the examination?

Your data will be kept for 15 years; this period is required by law.

10. What do we do with your data?

Are you participating in the research? Then you also give permission to collect, use and store your data.

What data do we keep?

We keep this data:

- your consent form for participating in the study

- anonymized data about your health

- (medical) data that we collect during the research, including the questionnaires

Why do we collect, use and store your data?

We collect, use and store your information to answer the questions of this study.

How do we protect your privacy?

To protect your privacy, we work with anonymized data. We put an anonymous code on all your documents. We keep the key to the code in a secure place in your hospital. When we process your data, we always use only that code. Also in reports and publications about the investigation, no one can recall that it was about you.

Who can see your data?

Some people in your hospital may have access to all your data. Also to the data without code. This is necessary to be able to check whether the research has been carried out properly and reliably. Persons who have access to your data for inspection are: your practitioner / researcher at your hospital, national supervisory authorities, such as the Health Care and Youth Inspectorate and research employees of the

Integraal Kankercentrum Nederland (IKNL) and the Clinical Trial Center Maastricht (CTCM). They keep your details secret. We ask you to give permission for this inspection.

May we use your data for other research?

After this study, your data may also be important for other scientific research in the field of pain after lung surgery. In the consent form, you indicate whether you are happy with your anonymous data being used for this purpose. You also indicate whether you may be approached for further research. Do you not give permission? Then you can still participate in this survey. You get the same care.

Can you withdraw your consent to the use of your data?

You can withdraw your consent to the use of your data at any time. This applies to use in this study and to use in other studies.

Would you like to know more about your privacy?

• Would you like to know more about your rights when processing personal data? Then look at www.autoriteitpersoonsgegevens.nl.

• Do you have questions about your rights? Or do you have a complaint about the processing of your personal data? Please contact the person responsible for the processing of your personal data. For your research that is:

o See Appendix A for contact details and website.

• If you have complaints about the processing of your personal data, we recommend that you first discuss them with the research team. You can also go to the Data Protection Officer of the Máxima Medical Center. Or you can submit a complaint to the Dutch Data Protection Authority.

Where can you find more information about the research?

For more information about the study, visit the following website: www.optrial.com

11. Will you be reimbursed if you participate in the study?

You will not be reimbursed if you participate in this study. There are no costs for the participants to participate.

12. Are you insured during the investigation?

An insurance policy has been taken out for everyone who participates in this study. The insurance pays for damage caused by the investigation. But not for all damage. In Appendix B you will find more information about the insurance and the exceptions. It also states who you can report damage to.

13. We do not inform your general practitioner and/or treating specialist

Since pain relief for lung surgery is a standard part of the care, the researcher will not inform your general practitioner and/or treating specialist about your participation in the study, but about the usual care.

14. Do you have any questions?

You can ask questions about the research to the researcher Louisa Spaans. Would you like advice from someone who has no interest in it? Then inquire with surgeon Mark van den Baar. He has knowledge about the investigation, but is not cooperating in this investigation. Appendix A contains contact details.

15. How do you give permission for the research?

You can think about this research first. Then, during your outpatient visit, you tell the lung surgeon whether you understand the information and whether or not you want to participate. Would you like to participate? Then fill in the consent form that you will find with this information letter (appendix C). You and the researcher will both receive a signed version of this consent form.

Thanks for your time.

Appendix A: information about the insurance

The sponsor (Máxima MC) has a covering insurance for everyone who participates in this research stduy. The insurance will pay for the damage that you have suffered as a result of taking part in the study. This concerns damage that you receive during the investigation, or within 4 years after the investigation. You must report damage to the insurer within 4 years.

Have you suffered damage as a result of the investigation? Report this to: e-mail louisa.spaans@mmc.nl or call 040-888-7243

The insurer of the investigation is:

Name: MediRisk

Address: Van Deventerlaan 20, 3528 AE Utrecht

Phone number: 030-202-7200

Email: info@medirisk.nl

Policy number: AB-1000103

The insurance pays a maximum of € 750,000 per person and € 5,000,000 for the entire research and € 7,500,000 per year for all scientific research carried out by or on behalf of the policyholder in that year.

Please note: the insurance does not cover the following damage:

• Damage due to a risk about which we have given you information in this letter. But this does not apply if the risk turned out to be greater than we previously thought. Or if the risk was very unlikely.

• Damage to your health that would also have occurred if you had not taken part in the study.

• Damage caused by you not or not properly following directions or instructions.

• Damage to the health of your children or grandchildren.

• Damage caused by a treatment method that already exists. Or through research into a treatment method that already exists.

These provisions are set out in the 'Compulsory insurance for medical research involving humans 2015 Decree'. This decision can be found in the government's Wettenbank (https://wetten.overheid.nl).

Appendix B: Subject Consent Form

OPtriAL: optimal postoperative pain management after lung surgery

− I have read the information letter. I could also ask questions. My questions have been answered well enough. I had enough time to decide whether to participate.

− I know that participation is voluntary. I also know that I can decide at any time not to participate in the study. Or to stop. I don't have to say why I want to stop.

− I am aware that the staff of the research team, the members of the review committee that approved the study, research staff from Integraal Kankercentrum Nederland (IKNL) and CTCM, national and international supervisory authorities have access to the original medical data and research data . I also understand that the research data in coded form can be used for scientific research and publications

− Would you like to tick yes or no in the table below?

I give permission to keep my data to use it for other research, as stated in the information letter.	Yes ☐	No☐
I give permission to ask me after this study if I want to participate in a follow-up study.	Yes ☐	No☐
I would like to be informed about the results after the study has been completed. The email address where I would like to be informed is:…………………………………..	Yes ☐	No☐

- I want to participate in this research study

My name is (study subject): ………………………………..

Signature: ……………………… Date : __ / __ / __

I declare that I have fully informed this subject about the abovementioned study. Will information become known during the study that could influence the subject's consent? Then I will let this subject know in time.

Name researcher (or their representative):……………………………….

Signature:……………………… Date: __ / __ / __

List of participating centers

Principal Investigator:

Máxima Medical Centre, Veldhoven

F.J.C. van den Broek, MD, PhD

E-mail: frankvanden.broek@mmc.nl

Local investigator(s):

Radboud University Medical Center Nijmegen

Marieke Kuut, MD

E-mail: Marieke.Kuut@radboudumc.nl

Spaarne Gasthuis, Haarlem

H. Rijna, MD, PhD

Email: rijna@spaarnegasthuis.nl

Zuyderland Medical Center, Heerlen

E. de Loos, MD

E-mail: e.deloos@zuyderland.nl

Medisch Spectrum Twente, Enschede

E.B. van Duyn, MD

E-mail: E.vanDuyn@mst.nl

Catharina Ziekenhuis Eindhoven

R. van den Broek, MD

E-mail: renee.vd.broek@catharinaziekenhuis.nl

Bravis Ziekenhuis, Bergen op Zoom & Roosendaal

D. Susa, MD, PhD

E-mail: d.susa@bravis.nl

Ikazia Ziekenhuis Rotterdam

N. Hanneman, MD

E-mail: nm.hanneman@ikazia.nl

Algemeen Ziekenhuis Sint-Maarten, Mechelen, België

Valentin Noyez, MD

E-mail: valentin.noyez@gmail.com

Universitair Ziekenhuis Antwerpen

Jeroen Hendriks, MD, PhD

E-mail: Jeroen.Hendriks@uza.be

Amsterdam Universitaire Medische Centra

Chris Dickhoff, MD, PhD

E-mail: c.dickhoff@amsterdamumc.nl
